# Enhancement of superconducting properties and flux pinning mechanism on Cr_0.0005_NbSe_2_ single crystal under Hydrostatic pressure

**DOI:** 10.1038/s41598-018-36672-x

**Published:** 2019-01-23

**Authors:** S. Arumugam, Manikandan Krishnan, Kent Ishigaki, Jun Gouchi, Rukshana Pervin, G. Kalai Selvan, Parasharam M. Shirage, Y. Uwatoko

**Affiliations:** 1 Centre for High Pressure Research, School of Physics, Bharathidasan University, Tiruchirappalli, 620024 India; 2 Institute of Solid State Physics, University of Tokyo, 5-1-5 Kashiwanoha, Kashiwa, Chiba, 277-8581 Japan; 3 Discipline of Metallurgy Engineering and Materials Science & Physics, Indian Institute of Technology Indore, Simrol Campus, Khandwa road, Indore, 453552 India; 4 Department of Physics, University of Alabama at Birmingham, Birmingham, AL 35294 USA

## Abstract

Superconducting properties of Cr_0.0005_NbSe_2_ (*T*_*c*_~6.64 K) single crystals have been investigated through the temperature dependent resistivity (~8 GPa) and DC magnetization (~1 GPa) measurements. Further, the critical current density (*J*_*c*_) as a function of applied magnetic field has been studied from magnetic isotherms. The vortex pinning mechanisms have also been systematically analyzed using weak collective pinning theory as a function of pressure. The *J*_*c*_ corresponds to the flux flow enhanced by the application of pressure due to increase of *T*_*c*_ and vortex changes. We found that the pressure is responsible for the spatial variations in the charge carrier mean free path (*δl* pinning). We find that core point pinning is more dominant than surface pinning which is caused by the application of pressure. In addition, *J*_*c*_*(H* = 0*)* increases from 3.9 × 10^5^ (0 GPa) to 1.3 × 10^6^ (1.02 GPa) A/cm^2^ at 2 K as the pressure is increased from normal pressure to 1.02 GPa. The pressure dependence of *T*_*c*_ (d*T*_c_/d*P)* becomes 0.91 K/GPa and 0.75 K/GPa from magnetization and resistivity measurements respectively. We found that the pressure promotes the anisotropy nature, and decrease of coherence length and resulting in pathetic interface of the vortex core with pinning centers.

## Introduction

Superconductivity in the transition metal dichalcogenides (TMDs) and their intercalated layered structure compounds have special features associated with extreme anisotropy of the superconducting materials^1^. Superconductivity and density waves are competing orders that derive from instabilities^2^ due to internal and external perturbations such as chemical pressure, external pressure and magnetic field. Spontaneous formation of periodic lattice distortions and Charge Density Waves (CDW) could be thermodynamically favorable under certain conditions in low dimensional metals where the wave vector is generally known to depend on the nesting properties of the Fermi surface^3^. The symmetry breaking suggest that phonon−electron coupling usually occurs at certain transition temperature (*T*_CDW_) and so called Peierls phase transition^2^. Recently, the TMDs are natural layered materials provided with a new platform to study superconductivity due to the tunable nature of the superconducting properties and coexistent with other collective electronic excitations as well as strong intrinsic spin-orbit coupling. The bulk crystals of TMDs are formed of monolayers and bound to each other by van der Waals attraction, which makes it feasible to investigate experimentally. Niobium diselenide (NbSe_2_) is one of the most studied layered TMDs which has van der Waals attraction between the layers and it has generated much attention due to interplay of superconducting transition temperature (*T*_c_)^4–7^ and *T*_CDW_^2,8–10^ is around 7 K and 33 K respectively. Anisotropy in TMDs, particularly in NbSe_2_ compounds, having the highest *T*_*c*_ among TMDs, could be significantly enhanced by introducing foreign atoms or molecules in the interlayer space (intercalation process)^6,9,11,12^. The intercalation ability of these compounds is related to the expected implementation of high temperature superconductivity in the sandwich type structures described by the excitonic mechanism^6,9,13^. Further remarkably, Zeeman-protected Ising superconductivity is expected in NbSe_2_ due to non-centrosymmetric structure with in-plane inversion symmetry breaking and strong spin-orbit coupling and the anomalous large in-plane critical magnetic field has become one important direction in crystalline 2D superconductors^11,12,14,15^. The vortex movement (i.e., vortex entry into or exit from a single-crystalline superconductor) is possibly pinned at the edges when applied very low magnetic field which can be generally attributed to the translational symmetry breaking at the edges and it is dependent on both shape and dimension of the superconductors^13,16^. The applied magnetic field would significantly modify the pinning and subsequently influence the magnetic behavior of the samples.

The TMDs superconductors have revealed wonderful superconducting properties including high values of *T*_c_, critical current density (*J*_c_), upper critical field (*H*_c2_) and irreversible field (*H*_irr_). In the presence of strong pinning, the vortex state of type-II superconductors is usually characterized by *J*_c_ that decreases monotonically with an increasing field (*H*) or temperature (*T*). In the weakly pinned superconductors, interplay between intervortex interface and flux pinning produces an unusual peak in *J*_c_ as a function of both field and temperature which are just below the normal-state boundary and it is usually designated as secondary peak effect^17,18^. Both strong pinning and high *J*_*c*_ depend on variation in the grain size and the coherence length (*ξ*)^19^. Vortex pinning arises from the interplay of several competing energies, namely the self-energy of the flux lines, vortex-vortex interactions, vortex inhomogeneity interactions and thermal excitations^20^. A magnetic field generates an array of vortices in type-II superconductors and the vortices strongly interact with each other forming highly correlated configurations such as the vortex lattice. In high-T_c_ cuprates at relatively high temperatures, vortices move and vibrate due to thermal fluctuations to the extent that the lattice can melt becoming a vortex liquid^21–23^. Growth of the structure of the vortex lattice in a weakly pinned high-T_c_ superconductor is of paramount importance, since it determines superconducting properties that are directly suitable for applications^24,25^. As the temperature is raised, the vortex lattice undergoes a first-order transition to a stable disordered state^24,26–28^. A thermal fluctuation permits pinned vortices to fluctuate around the potential energy and reduces the effective pinning energy due to thermal smearing. The vortices also escape totally from the pinning centers through a variety of de-pinning excitations. Vortex motion still occurs for currents lower than *J*_c_ at a much slower rate. This flux creep mechanism implies a residual dissipation and it is responsible for the time relaxation of persistent currents flowing in a superconducting closed loop.

Hydrostatic pressure effects on the *T*_c_ enhancement shows more advantages that are relevant to the flux pinning compared to other perturbations. The application of high pressure leads to changes in the electronic bands leading to original properties, which may be associated with a structural phase transition. It always reduces the lattice parameters and causes the shrinkage of unit cells, giving rise to the reduction of anisotropy. Grain connectivity improvement should also be expected, as pressure can compress both grains and grain boundaries. The formation of point defects can be more favorable under high pressure, since it is well known that the formation energy of point defects decreases with an increasing pressure. High pressure can cause low-angle grain boundaries to migrate in polycrystalline bulk samples, resulting in the emergence of giant grains, sacrificing surface pinning thereafter. Hence, a ratio of point pinning centers to surface pinning centers is expected to be higher due to an increase of formation energy under high pressure. Several examples can be recalled here. Pressure increases the superconducting transition from 3.5 K to 6.5 K and also the semiconducting to metallic transition in LaO_0.5_F_0.5_BiS_2_ single crystals^29^. Whereas a large enhancement of *T*_*c*_ from 26 K (0 GPa) to 43 K (4 GPa) in LaO_0.95_F_0.05_FeAs^30^ with application of external hydrostatic pressure up to 3 GPa using piston cylinder pressure device, *T*_*c*_ was reduced with the application of pressure above 3 GPa to 30 GPa, using a diamond anvil pressure device. Further, pressure induced superconductivity has been observed in the pnictides such as LaFeAsO_1−x_F_x_, LaFePO and SrFe_2_As_2_^31^. In the ladder compounds, pressure induces the metal-insulator transition generating hole carriers and eventually superconductivity occurs in Ca_14−x_Sr_x_Cu_24_O_11_^32^. Pressure is also an effective approach to improve the *J*_*c*_ significantly in FeSe^33^, Sr_4_V_2_O_6_Fe_2_As_2_^34^ YBa_2_Cu_3_O_7−x_^35^ superconductors, as the pressure induces more point pinning centers and subsequently affects the pinning mechanism^33,34^. The investigation of the pressure dependence of thermodynamic magnitudes proved to be a useful method of studying the properties of anisotropic compounds. Such investigations were performed on the layered compound NbSe_2_^36–39^. Pressure not only enhanced *T*_*c*_ with a rate of *dT*_*c*_*/dP* is 0.86 K/GPa (NbSe_2_) and 1.47 K/GPa (Fe_0.0011_NbSe_2_) but also increased *J*_*c*_ and *H*_*c2*_^38^. In the case of the intercalated NbSe_2_ pressure increased *T*_*c*_ and simultaneously suppressed *T*_*CDW*_^40^. Suderow *et al*., described the pressure dependence of anisotropy, of the electron-phonon coupling and Fermi velocities, which influence the peculiar interplay between CDW, Fermi surface complexity and superconductivity in NbSe_2_^41^. Pressure induces a transition from spatial variation in the *δT*_*c*_ pinning to *δℓ* pinning mechanism in the undoped NbSe_2_ superconductors^38^.

From above, it is clear that high pressure is exclusively unique in fine-tuning superconducting states with the benefit of without introducing disorder effects in comparison with chemical doping. High pressure is, thus, an important tool to study intrinsic properties of the materials to understand the enhancement of *T*_c_, *J*_c_ and other physical properties^23,33,34,36,38,40,42–44^. These facts motivated our present study on the pressure effects on the superconducting properties of single crystalline Cr_x_NbSe_2_. We anticipated that hydrostatic pressure would increase the superconducting volume, *H*_irr_, and *H*_c2_ due to enhancement of *T*_c_, increase the point defect and reduction in the anisotropy of single crystalline Cr intercalated NbSe_2_ samples. Indeed, we observed such interesting properties when pressure was applied to Cr intercalated NbSe_2_ is investigated. These findings are reported below.

## Results and Discussions

**Figure 1:**
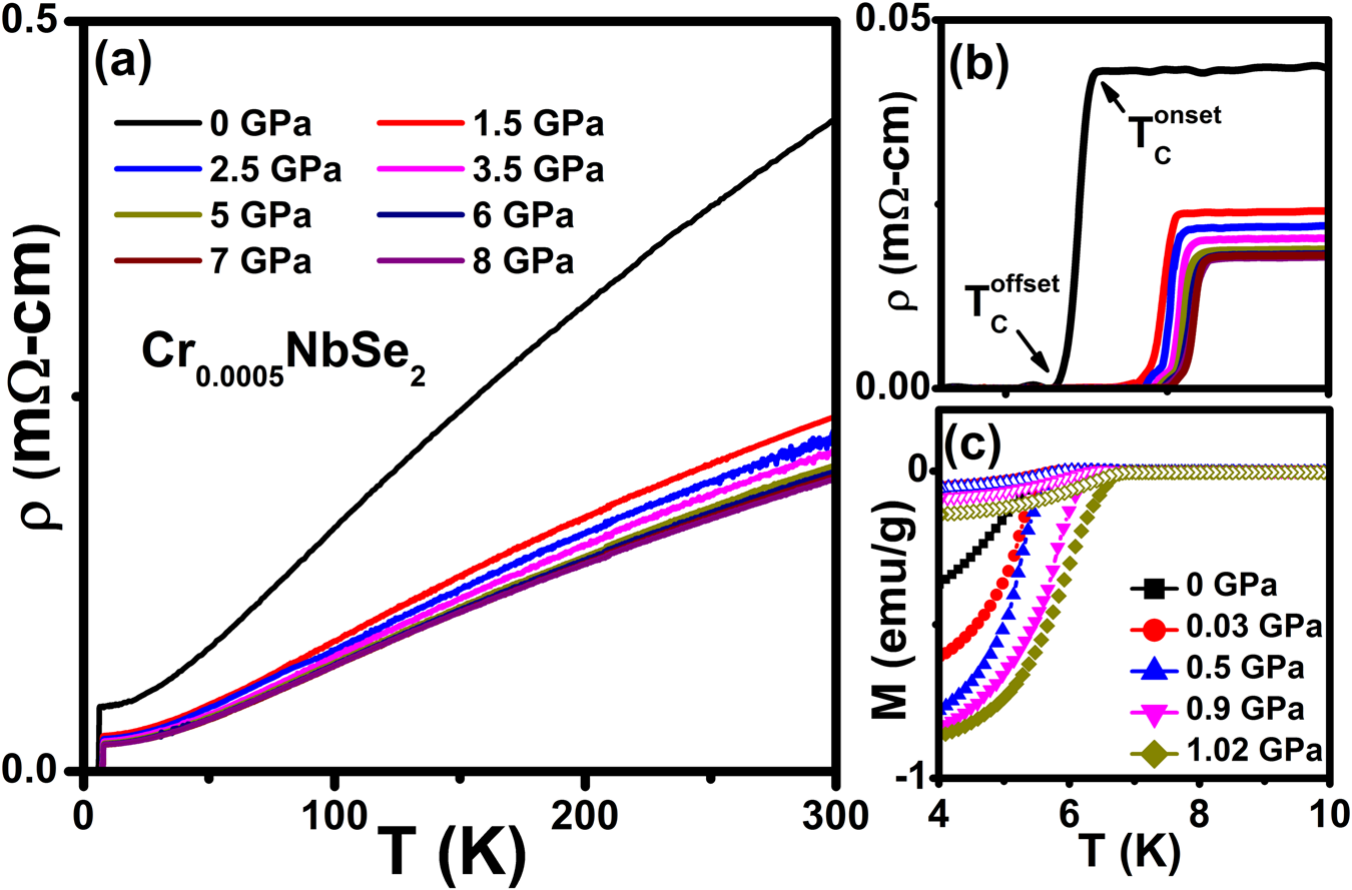
(**a**) Temperature dependent of resistivity at various applied hydrostatic pressures from 0 to 8 GPa, (**b**) pressure evolution of onset (*T*_c_^onset^) and offset (*T*_c_^offset^) superconducting transition temperature from resistivity measurements c) temperature dependent of zero field cooled (ZFC) [closed symbols] and field cooled (FC) [open symbols] dc magnetization at various hydrostatic pressures from 0 to 1.02 GPa.

The temperature dependence of resistivity (ρ(*T*)) in the temperature range from 2 to 300 K under various hydrostatic pressures from 0 to 8 GPa [Fig. 1(a)], the expanded region near *T*_c_ [Fig. 1(b)] and zero-field-cooled (ZFC) and field-cooled (FC) dc magnetization (*M*(*T*)) at the constant magnetic field of 20 Oe [Fig. 1(c)] in the vicinity of the T_c_ under various hydrostatic pressures range from 0 to 1 GPa of the crystal flakes of Cr_0.0005_NbSe_2_ are shown respectively in Fig. 1(a,b and c). The *T*_c_ is defined as an onset, mid and offset of superconductivity with obtained from dR/dT in *ρ(T)* measurements and the onset of diamagnetic signals corresponds to superconductivity as observed from *M*(*T*) measurements. As shown in Fig. 1(a), the *ρ* decreases (0.44 to 0.04 mΩ-cm at 0 GPa and 0.20 to 0.018 mΩ-cm at 8 GPa) in the temperature region from 300 to 10 *K*. It is also clear from Fig. 1(c), that *M*(*T*) starts to deviate from the normal state behavior around 6.1 K due to the presence of a diamagnetic signal, which is slightly above the onset of *T*_c_. The sharp superconducting transition along with the large residual resistivity ratio (~11) and superconducting width $({\rm{\Delta }}{T}_{c}={T}_{c}^{onset}-{T}_{c}^{offset})$ indicate good quality of single crystals used in the present study. The observation of sharp superconducting transition and zero resistivity at each applied pressure ensure indicate the hydrostatic nature in our experiments. As shown in Fig. 1(a), normal state resistivity gradually decreases with the application of high pressure and it almost shows the metallic nature for all pressures up to ~ 8 GPa. Similar *ρ(T)* behavior under pressure has been reported for various superconducting materials^33,34,36,38,40^. This is associated with the fact that high pressure brings the layers in the unit cell closer together, and facilitates overlap of wave functions of the conduction electrons in the adjacent layers. The *T*_*c*_ (6.64 K) of Cr_0.0005_NbSe_2_ is found to be less than undoped NbSe_2_ single crystal, and similar trend has been reported for various intercalated compounds Fe_x_NbSe_2_^6,38^, NbSe_2_^36,40^, Ga_x_NbSe_2_^11^, Pd_x_NbSe_2_^45^, Cu_x_NbSe_2_^12^, (LaSe)_1.14_(NbSe_2_)^46^ pnictides^34,44^ at both ambient and pressure.

**Figure 2:**
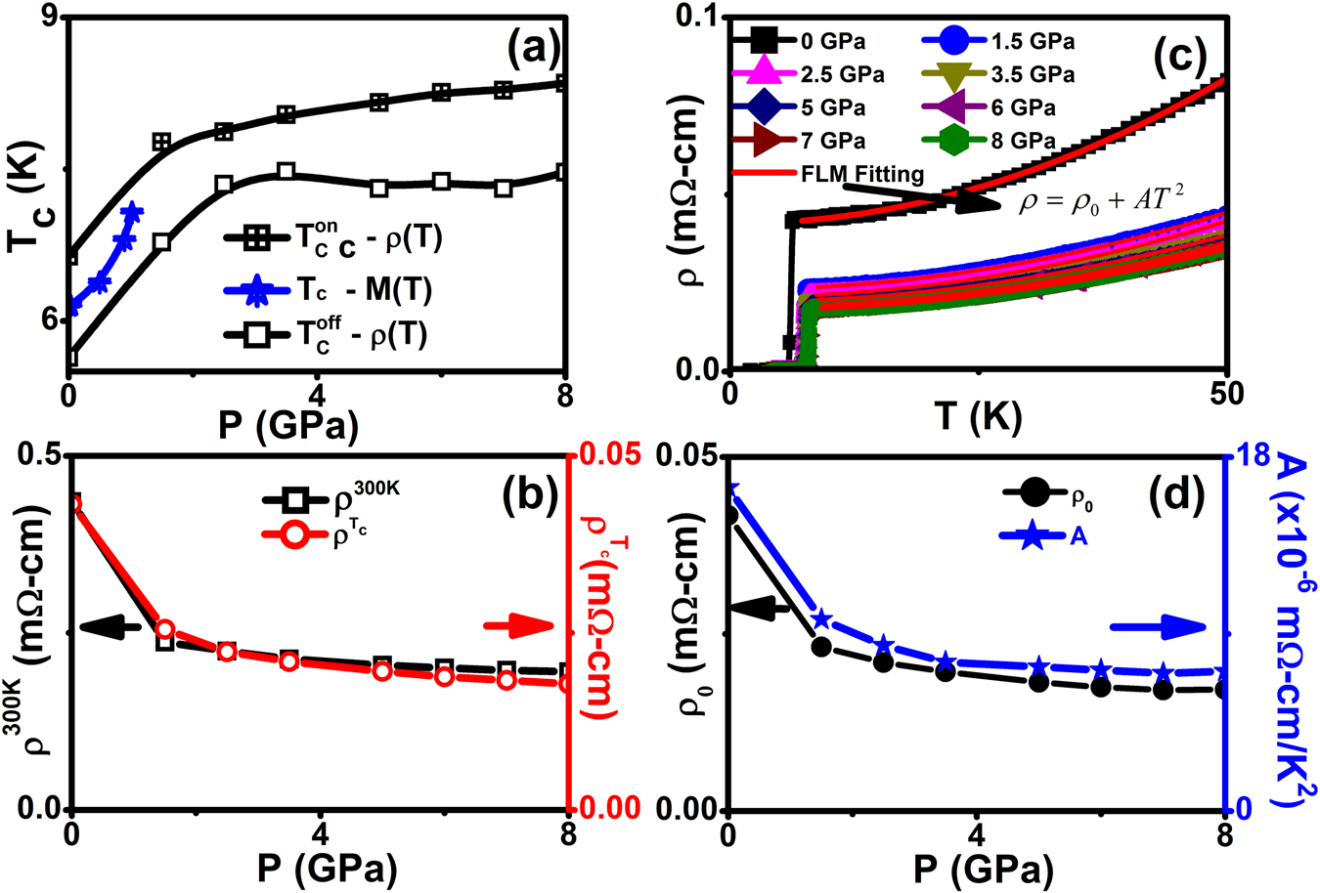
(**a**) Pressure dependent of superconducting transition temperature (*T*_c_) from resistivity and magnetization measurements, (**b**) pressure dependent of resistivity of *T*_c_^onset^ (ρ^*T*^_c_) and room temperature (ρ^300K^), (**c**) excellent fitting to the resistivity data from 7 to 50 K using Fermi-liquid model (*ρ* = *ρ*_0_ + *AT*^ 2^) for various pressures, and (**d**) pressure dependent of residual resistivity (ρ_0_) and scattering factor (*A*) from FLM fitting for Cr_0.0005_NbSe_2_.

Figure 1(c) shows *M*(*T*) in the ZFC and FC regimes with an applied magnetic field of 20 Oe under various hydrostatic pressures up to ~ 1 GPa in the vicinity of T_c_ up to 10 K; We clearly notice the diamagnetic transition at 6.14 K and 7.07 K at ambient and 1.02 GPa respectively. The diamagnetic transition shifts towards high temperature with the application of pressure as shown in Fig. 2(a). The hysteresis between ZFC and FC regimes indicates that Cr_0.0005_NbSe_2_ exhibits weak flux pinning centers. Without correcting the demagnetization factor, we estimate the superconducting volume fraction (ZFC) to be ~ 80% indicative of bulk superconductivity. At ambient pressure the sample shows a *T*_c_ of 6.14 *K* from *M*(*T*) at constant magnetic field of 20 Oe and it is ∼0.5 K less than that observed from ρ(*T*) measurements, as shown in Fig. 2(a). Evidently, ${T}_{c}^{onset}$ and ${T}_{c}^{offset}$ observed from *ρ(T)* is higher than *M*(*T*) measurements as shown in Fig. 2(a). With the application of high pressure, *T*_c_ onset steadily increases in entire pressure region due to the reduction of interlayer distances of Cr_0.0005_NbSe_2_ as shown in Fig. 2(a).

With an application of pressure, the room temperature resistivity (*ρ*^*300K*^) decreases monotonically up to 8 GPa and favours enhancement of metallic nature [Fig. 1(a)]. However, ρ^300K^ decreases at a faster rate (0.13 mΩ-cm/GPa) up to 1.5 GPa and then moderate decrease (0.01 mΩ-cm/GPa) is observed above 1.5 GPa to 8 GPa. Further, ${\rho }^{{{\rm{T}}}_{{\rm{c}}}({\rm{o}}{\rm{n}}{\rm{s}}{\rm{e}}{\rm{t}})}$ decreases with slower rates are 0.01 mΩ-cm/GPa and 0.001 mΩ-cm/GPa at below and above 1.5 GPa respectively. Hence, we found that both ρ^300K^ and ρ^*T*c(onset)^ are sensitive to the low pressure as shown in Fig. 2(b). *ρ(T)* is found to be almost linear in the high temperature region for various applied pressures. The change in ρ(*T*) shows metallic behavior at all pressures of up to 8 GPa. The upward curvature in ρ(*T*) weakens progressively with increasing pressure. Figure 2(a) shows the rapid enhancement of *T*_*c*_ in the low pressure region and moderate increase in high pressure region as we observed from both ρ(*T*) and *M*(*T*) for Cr_0.0005_NbSe_2_. One can see that the both *T*_c_^onset^ and *T*_c_^offset^ shift towards higher temperature with the application of high pressure and the d*T*_c_/d*P* becomes 0.91 K/GPa in the 0 < *P* < 1 GPa from *M*(*T*) measurements. The rate of change of *T*_c_ with pressure d*T*_c_/d*P* = 0.75 K/GPa for P less than 1.5 GPa and 0.09 K/GPa in the interval 1.5 < *P* < 8 GPa as observed from ρ(*T*) measurements. These results suggest that the relative change in the density of states at the Fermi level is profound in low pressure region up to 1.5 GPa and the moderate increase in the high pressure region (1.5 < *P* < 8 GPa) which leads to corresponding variation of *T*_c_. This is associated with the fact that the *P* brings the layers closer together, and it facilitates the overlap of the wave functions of the conduction electrons in the adjacent layers. Consequently, an increase in *T*_c_ is mainly determined by changes in the density of states at the Fermi level and the change in the phonon spectrum plays a minor role. The region above 100 K in Cr_0.0005_NbSe_2_ can be described reasonably well by straight line fits with different slopes for various pressures in ρ(*T*) suggesting that phonon scattering mechanism is dominant.

The relationship between interlayer spacing and *T*_*c*_ is a matter of fundamental importance in understanding of superconductivity in layer compounds. The effect of pressure upon the physical properties is due to the reduction in interlayer spacing. The band structure calculations of the Fermi level for NbSe_2_ at the middle of a narrow d sub-band situated between the unoccupied and primarily d bands of Nb and the fully occupied p bands of Se. Although differing in the details of the sub-band overlap with the p bands, both calculations highlight the importance of interband (hybridization) and interlayer interactions. These take the form of a nonzero empty site potential at the unoccupied interstitial positions between the Se layers due to wave function overlap in the modified muffin tin potential approach of Kasowski^47^. From earlier report, we inferred that neglecting a weak empty site potential broadens the sub-band and increases the overlap with the p bands. If the charge transfer occurs due to intercalation, shifts in the Fermi level towards the sloped region of the density of states curve and this can lead to a relatively a big change in density of states (*N*(ε_F_)) under pressure, and consequently, in *T*_c_. More detailed information about the electronic properties and superconductivity of the pure and intercalated NbSe_2_ solid solution was obtained from specific heat^12^ measurements.

The phonon mediated superconductors, the electron-phonon coupling constant have been estimated from McMilllan formula^48^ using Debye temperature^49^ and T_c_,

$${T}_{c}=\frac{{\theta }_{D}}{{\rm{1.45}}}\exp (-\frac{{\rm{1.04}}({\rm{1}}+\lambda )}{\lambda -\mu ({\rm{1}}+{\rm{0.62}}\lambda )}),$$

where *θ*_*D*_ is the Debye temperature, µ^*^ is the screened pseudo-potential (characteristic for the electron repulsion) and assumed to be 0.15 suggested by McMilllan^48^ for transition metallic superconductors and *λ* is the electron-phonon coupling constant and the parameters *θ*_*D*_, µ^*^ and *λ* are all pressure dependent. The values of *λ* is 0.84 at ambient pressure suggest that strong coupling in superconductivity. With the Sommerfeld parameter (*γ*) and the electron-phonon coupling constant (*λ*), the electron density of states at the Fermi level (N(ε_F_)) can be obtained from $N({\varepsilon }_{F})={\rm{3}}\gamma /{\pi }^{{\rm{2}}}{k}_{{B}^{{\rm{2}}}}({\rm{1}}+\lambda )$. The density of electronic states at the Fermi energy therefore clearly decreases when more metal ions intercalates into NbSe_2_. All the values of *θ*_*D*_ and dθ_D_/d*P* for pure and intercalated samples are identical. The pressure dependence of Debye temperature θ_D_(*P*) can be obtained from the Gruneisen’s formula^48^, $\frac{{\rm{1}}}{{\theta }_{D}}=\frac{\partial {\theta }_{D}}{\partial P}=\frac{\alpha V}{{C}_{V}}$, where α is the thermal expansion coefficient, *C*_v_ is the heat capacity and the value of dθ_D_/d*P* is one order of magnitude lower than the corresponding changes is *T*_c_ with pressure. The large relative growth of *T*_c_ can be qualitatively associated with the particularities of the NbSe_2_ band structure, and corresponding changes of λ(*P*)^40^. Earlier it was shown that the intercalated Fe and Cu is located in the interlayer space, when the superconducting properties are affected by the interlaminar intercalant^12^. A redistribution of the interlayer under pressure can serve as an additional reason for the changes in *T*_c_^39^. The last idea is supported by an increase of the width of the superconducting transition under pressure and more pronounced manifestation of its stepped form. This type of evolution in the superconducting state under high pressure^38,44,50^ has been observed in layered structure superconductors with a small anisotropy parameter^23,33,34^.

It is known that *T*_c_ is found to increase for NbSe_2_^12,39^, Fe_x_NbSe_2_^38^ and Cr_0.0005_NbSe_2_ compounds by applying high pressure. Figure 2(c) examines the normal-state resistivity of Cr_0.0005_NbSe_2_ under hydrostatic pressure and its implication of electronic correlation. Low temperature region (7 ≤ *T* ≤ 50 K) of ρ(T) can be fitted using the Fermi liquid model, *ρ* = *ρ*_0_ + *AT *^2^, where ρ_0_ is residual resistivity and *A* is a scattering factor. Figure 2(c) shows good fitting for both data at ambient and high pressure and it supports the Fermi liquid model. The value of ρ_0_ is 0.042 mΩ-cm: 0 GPa and 0.017 mΩ-cm (8 GPa) and the A [16.45 × 10^−6^ mΩ-cm/K^2^ (0 GPa) and 7.1 × 10^−6^ mΩ-cm/K^2^: 8 GPa] value shows clear indication of electron-electron interaction exhibits in both ambient and high pressure in this sample. The nature *ρ*_*0*_ and *A* under various hydrostatic pressure has been shown in Fig. 2(d) which suggests that Cr_0.0005_NbSe_2_ is a weakly correlated system.

**Figure 3:**
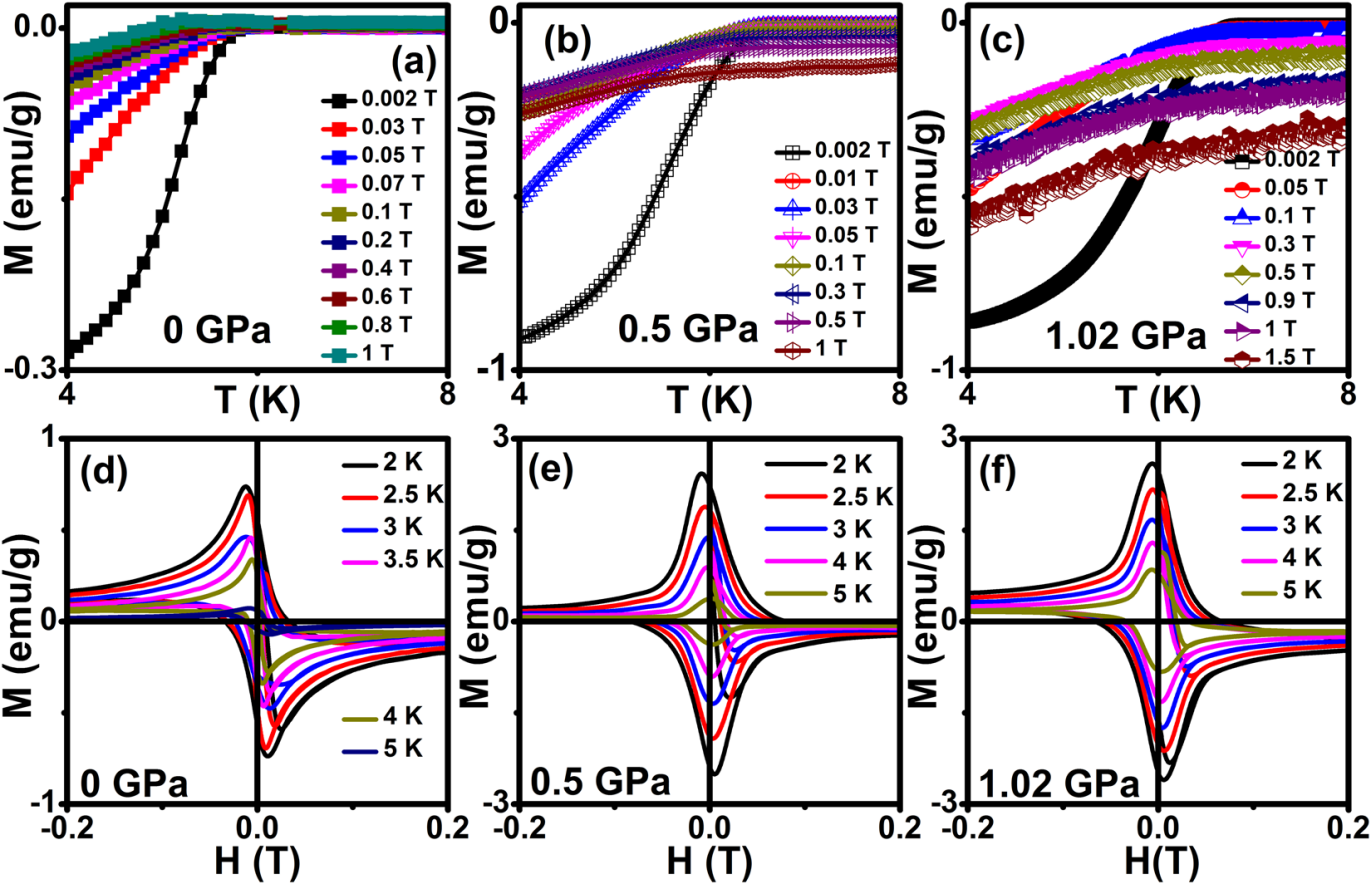
(**a**), (**b**) and (**c**) Temperature dependent of dc magnetization at various magnetic fields for 0, 0.5 and 1.02 GPa respectively on Cr_0.0005_NbSe_2_, (**d**), (**e**) and (**f**) Field dependent of magnetization scan in a low field range (d*H*/d*t* ~ 20 Oe/s) at various temperatures for 0 GPa, 0.50 GPa & 1.02 GPa respectively on Cr_0.0005_NbSe_2_.

Figure 3(a–c) [Fig. S2] shows the temperature dependent of dc magnetization with various applied magnetic fields (*H*) with the constant *P* of 0, 0.5 and 1.02 GPa respectively. It is found that both *T*_c_ and diamagnetic signal show decreasing trend and allows us to determine the upper critical field (*H*_c2_) of this material at various pressures. Taking the onset of transition in *M*(*T*) at various magnetic fields with constant pressure as the upper critical field point *H*_c2_(*T*_c_) and infer that almost all Cooper pairs are broken at this temperature and *H*. Further, the Meissner signal is suppressed with the application of various *H* at 0 GPa. However, the Meissner signal enhances at various *H* with constant pressure of 0.5 and 1 GPa, and it confirms that the pinning centers increase due to the application of pressure. Figure 3(d–f) shows the field dependent isothermal magnetization (MHL) scan in a low field region under various temperatures with applied pressure of 0, 0.5 and 1 GPa respectively. These results reveal that magnetic moment increases with the application of constant pressure, and confirms that when the occurrence of pinning increases as pressure increases in the sample.

According to the Ginzburg–Landau (GL) theory, the absolute zero temperature upper critical field *H*_c2_(0) can be estimated by using formula, *H*_*c*2_(*T*/*T*_*c*_) = *H*_*c*2_(0)(1 − [*T*/*T*_*c*_]^*a*^)^*b*^ with a = 1.39 and b = 1 associated with the large gaps that open in the Nb bands^51^ which gives values less than that observed from WHH approach and the values *H*_c2_(0) [Fig. S1] are shown in Table 1. The orbital limited upper critical fields [${H}_{c{\rm{2}}}^{orb}({\rm{0}})$] are estimated using the Werthamer–Helfand–Hohenberg (WHH)^52^ empirical formula, ${H}_{c{\rm{2}}}^{orb}({\rm{0}})=-\,{\rm{0.693}}{T}_{c}{(d{H}_{c2}/dT)}_{T={T}_{c}}$ and these values are shown in Table 1. For the Superconducting materials exhibiting weak coupling case, the Pauli limited upper critical field is calculated from, *H*_*p*_(0) = 1.84*T*_*c*_, where *T*_c_ is taken from *M*(*T*) measurements and the values are shown in Table 1. *H*_p_(0) indicating that both orbital effect and Pauli spin paramagnetic effect (PSP) which gives an influence on the pair-breaking mechanism through the entire pressure region. However, we haven’t excluded the effect of spin instability at low temperatures, which may very well play an important role in the occurrence of superconductivity. The absolute orbital upper critical field ${H}_{c{\rm{2}}}^{orb}({\rm{0}})$ [Fig. S1] destroys superconductivity characteristics by enhancing the pair breaking phenomena in the presence of magnetic field and the values of ${H}_{c{\rm{2}}}^{orb}({\rm{0}})$ are found to be higher at higher pressures than at ambient pressure. Further, the enhancement of critical field under pressure implies that the strong flux pinning exhibits in this sample. Since, Pauli limit superconductivity mechanism [${H}_{c{\rm{2}}}({\rm{0}}) < {H}_{c{\rm{2}}}^{orb}({\rm{0}}) < {H}_{p}({\rm{0}})$] is exhibited by this sample, the calculated Maki parameter $\alpha =\sqrt{{\rm{2}}}{H}_{c{\rm{2}}}^{orb}({\rm{0}})/{H}_{p}({\rm{0}})$, is found to be nearly 1 at both ambient and at high pressures. Similar pressure dependences of *H*_c2_(*P*) were reported in YBCO^42^, FeSe^43^ Fe_x_NbSe_2_^38^ indicating clearly that pressure influences the upper critical field. The superconducting coherence length [*ξ*_GL_(0)] can be estimated from the relation, *ξ*_*GL*_(0) = [*Φ*_*0*_/2*πH*_*c*2_(0)]^0.5^ where *Φ*_*o*_ = 2.07*X*10^−7^*G*−*cm*^2^ and the values of *ξ*_*GL*_(0) are calculated from ambient and high pressure and it is shown in Table 1.

**Table 1:** Summary of the upper and lower critical field for the Cr_0.0005_NbSe_2_ single crystal under pressure.

*P* (GPa)	*H*_*p*_(0) (T)	${{\boldsymbol{H}}}_{{\boldsymbol{c}}{\bf{2}}}^{{\boldsymbol{orb}}}({\bf{0}})$ (T)	*H*_*c*2_(0) (T)	*ξ*_*GL*_(0) (nm)	${{\boldsymbol{H}}}_{{\boldsymbol{c}}{\bf{1}}}^{{\boldsymbol{\text{'}}}}({\bf{0}})$ (T)	*λ*(0) (nm)
0	11.30	5.89	4.81	8.73	0.051	80.4
0.5	11.79	9.68	7.53	6.50	0.159	59.2
1.02	12.36	10.27	9.39	5.82	0.178	56

**Figure 4:**
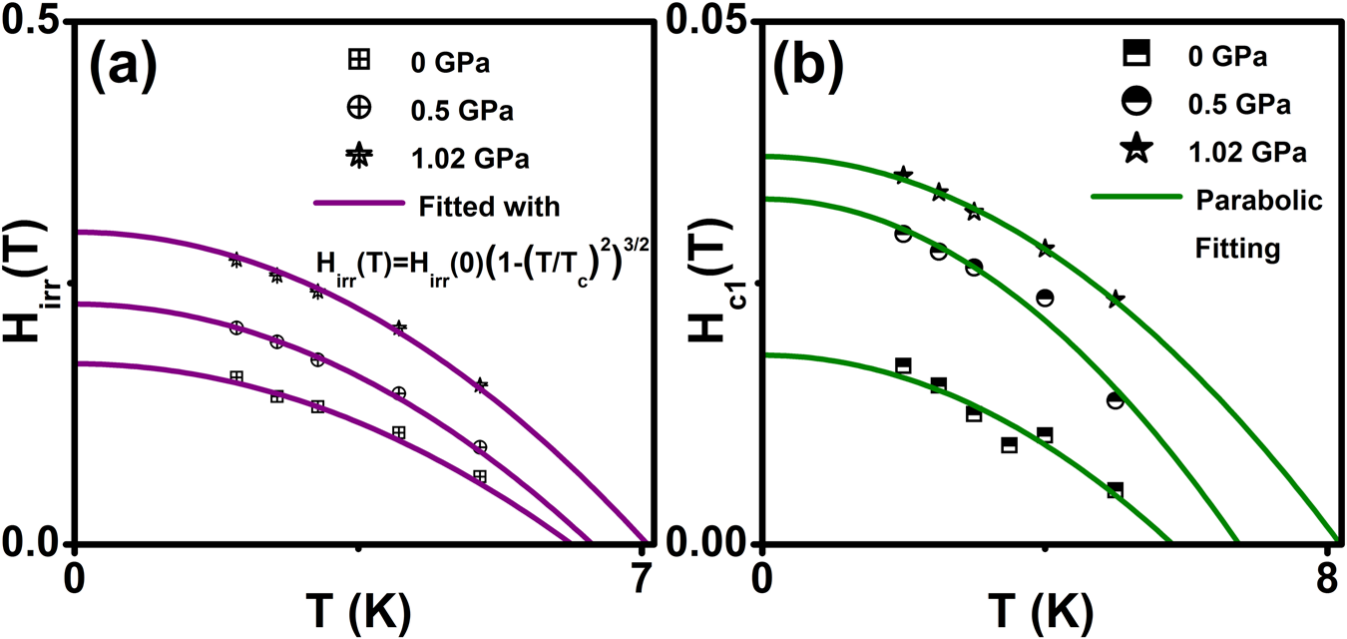
(**a**) Temperature dependence of irreversible field (*H*_irr_) for Cr_0.0005_NbSe_2_ sample under pressure. Solid lines represent how fit the relation *H*_*irr*_(*T*) = *H*_*irr*_(0)(1 − (*T*/*T*_*c*_)^2^)^3/2^, (**b**) shows the lower critical field (*H*_c1_) for Cr_0.0005_NbSe_2_ sample under pressure. Solid lines show the parabolic fitting.

The irreversible field (*H*_irr_) is calculated from MHL using the criteria of the zero field current density [*J*_c_(*H* = 0)] and it occurs due to depinning of the magnetic fluxes in this sample. Figure 4(c) shows temperature dependent of *H*_*irr*_ fitted with the equation, *H*_*irr*_(*T*) = *H*_*irr*_(0)(1 − (*T*/*T*_*c*_)^2^)^3/2^, pressure up to ~ 1.02 GPa. It reveals that enhancement of *H*_irr_ with application of pressure and also provides evidence of the 3D nature of flux creep in the sample. This is an indication that *H*_irr_ is mainly controlled by the flux pinning. *H*_c1_ is measured from MHL under various temperatures [Fig. 3(a–c)] and plotted as a function of temperature with the parabolic function fitting as shown in Fig. 4(d). The precise determination of *H*_*c1*_*(0)* from MHL possibly suffers from demagnetization effect. Further, *H*_c1_(0) can also be deduced from the first penetration field ${H}_{c{\rm{1}}}^{\text{'}}({\rm{0}})$, assuming that the magnetization *M* = −*H*_*c*1_, when the first vortex enters into the sample. Thus, magnetic field has rescaled to *H*_*eff*_ = *H*−*NM* and ${H}_{c{\rm{1}}}({\rm{0}})={H}_{c{\rm{1}}}^{\text{'}}({\rm{0}})/({\rm{1}}-N)$, where *N* is a demagnetization factor and H is a magnetic field. It has been shown by Brandt^53^ that a bar sample with a rectangle cross-section, the effective demagnetization factor $N={\rm{1}}-\,\tanh \,\sqrt{{\rm{0.36}}t/b}$, where *b* and *t* width and thickness of the sample. Using this criterion, *H*_c1_(0) was estimated and fitted to the parabolic temperature dependence *H*_*c*1_(*T*) = *H*_*c*1_(0)(1 − (*T*/*T*_*c*_)^2^) and the experimental *H*_c1_(0) is estimated at various pressures. The fit of our data to this expression (solid red line) suggests that vortex penetration in this material can be well described by BCS theory. Although the demagnetization effect is small and corrected lower critical field ${H}_{c{\rm{1}}}^{\text{'}}({\rm{0}})$ is calculated from *H*_c1_(0) with demagnetization correction using Brandt’s formula,${H}_{c{\rm{1}}}^{\text{'}}({\rm{0}})={H}_{c{\rm{1}}}({\rm{0}})/\,\tanh \,\sqrt{{\rm{0.36}}t/b}$ for approximate slab geometry^53^ and these values of ${H}_{c{\rm{1}}}^{\text{'}}({\rm{0}})$ listed in Table 1. Using the *H*_c1_(0), it is possible to estimate the penetration depth from the relation, $\lambda ({\rm{0}})={[(\Phi {}_{{\rm{0}}}/{\rm{2}}\pi {H}_{c{\rm{1}}}^{\text{'}}({\rm{0}}))(\mathrm{ln}\kappa +{\rm{0.5}})]}^{{\rm{0.5}}}$, where λ(0) is the penetration depth at 0 K. The ratio between the λ(0) and coherence length (*ξ*_GL_(0) gives GL parameter (κ) through the expression *κ* = *λ*(0)/*ξ*_*GL*_(0). The value of $\kappa =\sqrt{{\rm{1}}/{\rm{2}}}$ has been conventionally used to classify superconductors as type I or type II based on whether κ value is higher or lower than $\sqrt{{\rm{1}}/{\rm{2}}}$. Our analysis predicts that κ value is very much higher than critical value and indicates Cr_0.0005_NbSe_2_ as a type II superconductor. Similarly Cu_x_NbSe_2_^12^ and Zr_0.96_V_0.04_B_2_^54^ compounds were shown to be type-II superconductor, since κ value has been reported larger than the critical value.

**Figure 5:**
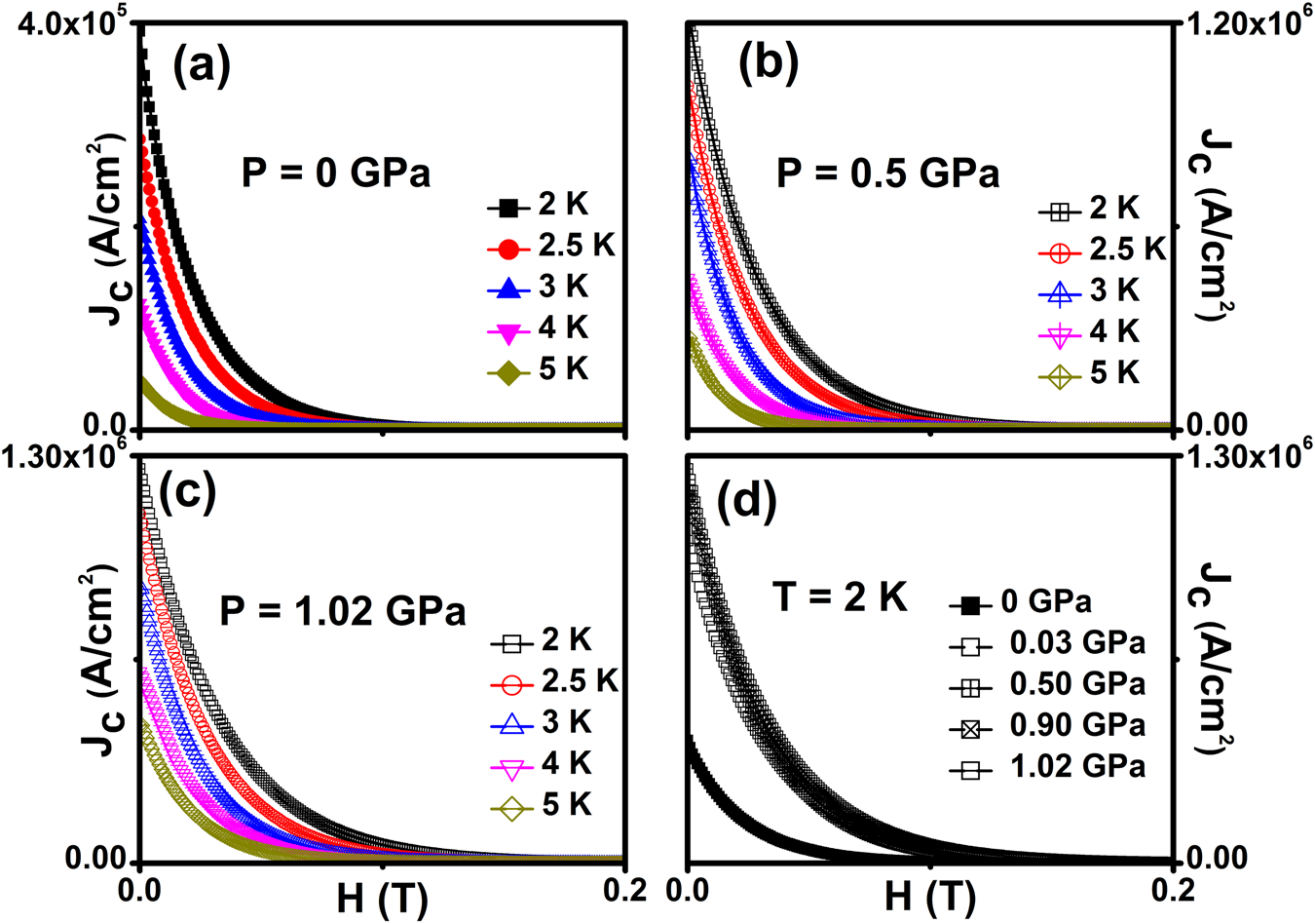
(**a**), (**b**), (**c**) and (**d**) Magnetic field dependent critical current density (J_c_(H) on Cr_0.0005_NbSe_2_ for various hydrostatic pressures at various temperatures.

Figure 5(a–d) shows the field dependence of superconducting critical current density (*J*_c_(*H*)) on Cr_0.0005_NbSe_2_ for various temperatures at constant hydrostatic pressures of 0, 0.03, 0.5, 0.90 and 1.02 GPa. The *J*_c_(*H*) is estimated from MHL using Bean’s model^55,56^ *J*_*c*_ = 20Δ*M*/(*b*(3*l* − *b*)/3*l*), where width of the magnetization [Δ*M* = *M* + (*H*) − *M*^−^(*H*)], b is a width and *l* is a length of the samples (*b* < *l*) were in mm. In general, low-magnetic field region, the gap Δ*M* in magnetization loop is mainly caused by the intergranular current. However, in the high-field region, Δ*M* results largely due to the intragranular current. Similar results have been reported from *M(H)* measurements on cuprates^57^ and pnictides^58,59^.

**Figure 6:**
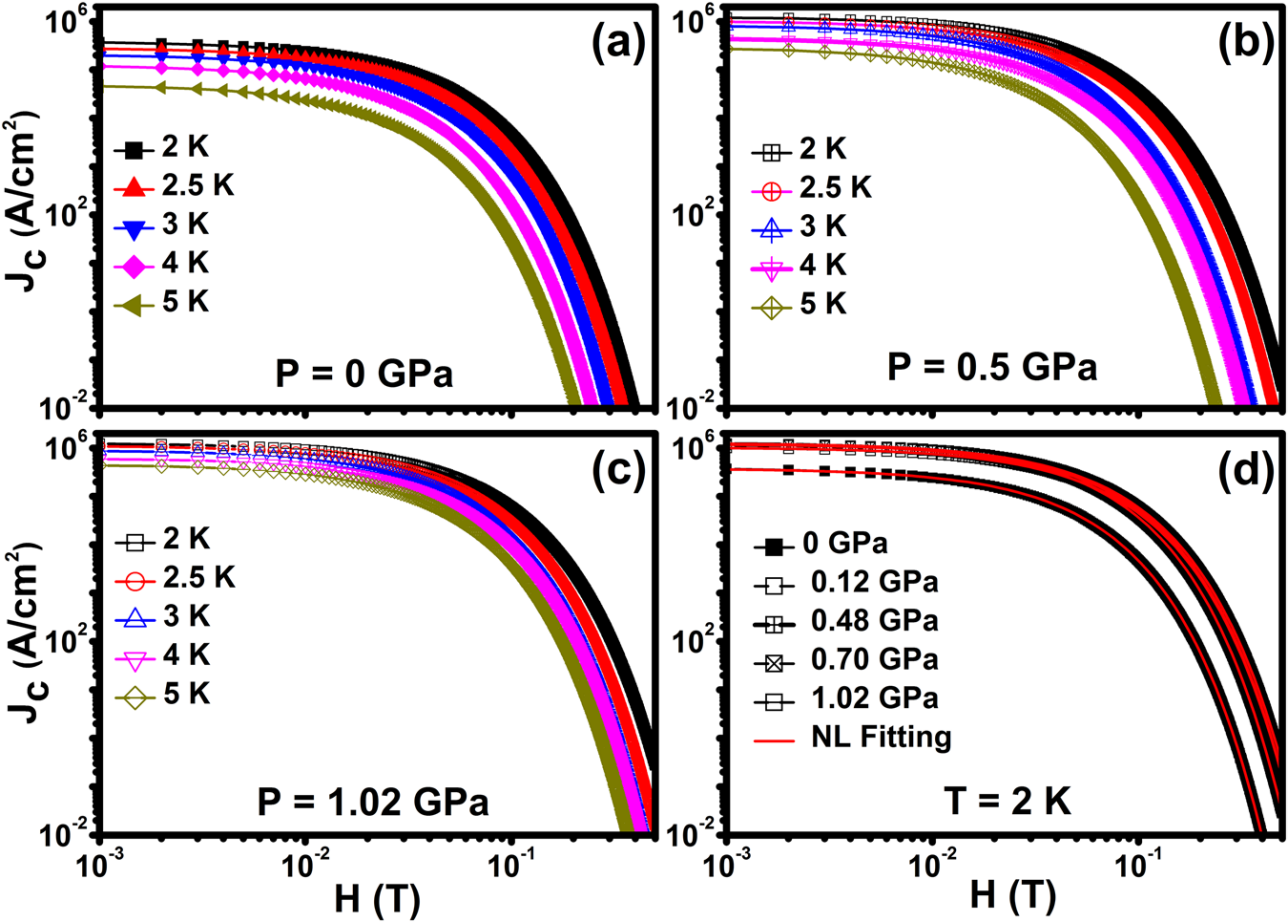
(**a**), (**b**), (**c**) and (**d**) Logarithmic scale of magnetic field dependent superconducting critical current density *J*_c_(H) on Cr_0.0005_NbSe_2_ for various temperatures and a few fixed hydrostatic pressures. Solid lines are shown in the collective pinning model fitting at the log scale at a temperature of 2 K.

High pressure enhances the *J*_c_ in Cr_0.0005_NbSe_2_ sample and subsequently enhances flux pinning with an increase of point pinning centers. The *J*_c_(0) values determined from MHL curves at 2 K are 391884, 1029067, 1190414, 1169989, 1258268 A/cm^2^ for 0, 0.03, 0.5, 0.9 and 1.02 GPa respectively and the *J*_c_(*H* = 0) increased by a factor of three at higher pressures. Figure 6(a–c) shows *J*_c_(*H*) [Fig. S3] in the logarithmic scale for various temperatures and reveals that *J*_*c*_ exponentially decreases as the H increases. This result indicates that occurrence of weak pinning singularities, since magnetic impurities (Cr) act as point pinning centers. Further Fig. 6(d) shows exponential decay of *J*_c_ with an increase in applied magnetic field and these results compared theoretically, *J*_*c*_(*H*) = *J*_*c*_(0)exp{−(*H*/*H*_0_)^3/2^} and found excellent fitting for various pressure and temperatures.

**Figure 7:**
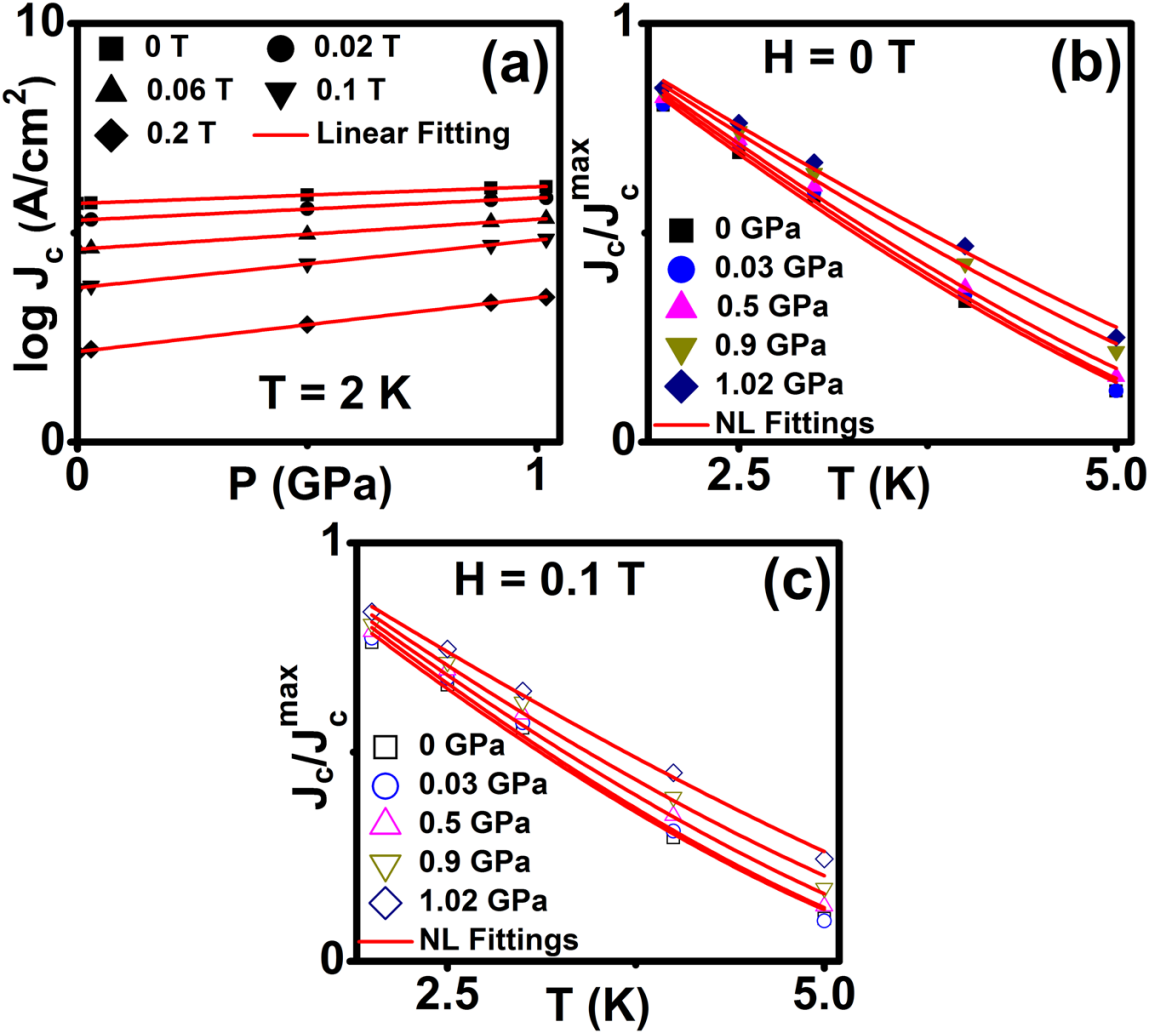
(**a**) Pressure dependence of *J*_c_ at 0, 0.02, 0.06, 0.1 and 0.2 T for 2 K. Solid lines are shown in the Linear fitting, (**b**) and (**c**) Temperature dependent normalized *J*_c_ at various pressures for constant magnetic field of 0.05 and 0.1 T. Solid lines are shown in the *J*_*c*_ ∝ (1 − *T*/*T*_*c*_)^*n*^ fitting.

Figure 7(a) shows *J*_c_ as a function of pressure in logarithmic scale at 2 K under various fields and the solid lines show linear fits to the data, which gives the slopes [d(log*J*_c_)/d*P*] of 0.40, 0.53, 0.71, 1.14 and 1.29 GPa^−1^ at 0, 0.02, 0.06, 0.1 and 0.2 T respectively. These results indicate that application of pressure leads to an enhancement in *J*_c_ and it is more significant at higher fields. The normalized *J*_c_ as a function of temperature at 0 and 0.1 T for various pressures is shown in Fig. 7(b,c) and fitted with the scaling relation *J*_*c*_ ∝ (1 − *T*/*T*_*c*_)^*n*^ where *n* is the critical exponent at each pressure. It is known that GL theory predicts distinct vortex pinning mechanisms in superconducting materials, with different values of exponent (*n*) at specific fields. The value of *n* = 1 and *n* >1.5 corresponds to non-interacting vortices and strong vortex core pinning mechanism respectively. The critical exponent is estimated from fitting the scaling relation in Fig. 7(b,c) for various magnetic fields at constant hydrostatic pressure. It reveals that the values of n are found to be 1.65 ≤ *n* ≤ 2.12(0 T) and 2.82 ≤ *n* ≤ 3.75 (0.1 T) under various hydrostatic pressure and shows higher value of n under pressure than at ambient pressure. Similar behaviour has been reported on J_c_(P), J_c_(T) and vortex dynamics properties in Fe_x_NbSe_2_^38^ and in pnictides^34^.

**Figure 8:**
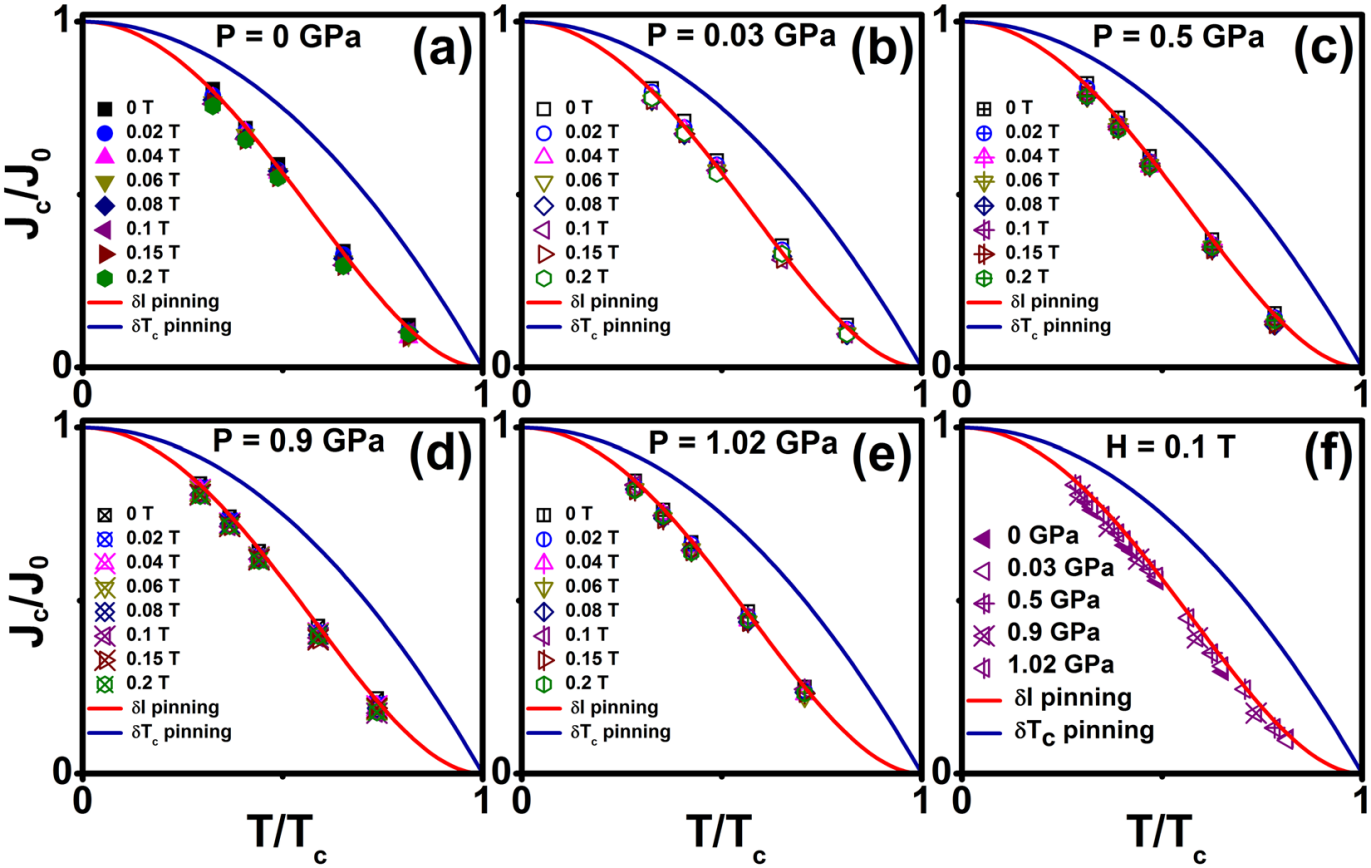
(**a**–**f)** Normalized *J*_c_ (*J*_c_/*J*_0_) as a function of reduced temperature (*T*/*T*_c_) for various hydrostatic pressures on Cr_0.0005_NbSe_2_. The Solid line (red) is shown in δ*l* pinning fitting and the solid line (blue) is shown in *δT*_*c*_ pinning fitting.

The core pinning mechanism is examined in the framework of collective pinning theory in this sample. Generally, core pinning comprises that pinning mechanism related to the spatial variation in the charge carrier mean free path (*l*), called *δl* pinning and pinning due to randomly distributed spatial variation in T_c_, called *δT*_*c*_ pinning, which is mostly due to crystal defects^60^. We analyzed the pinning mechanism as reported by Griessen *et al*.^60^ using the relation *J*_*c*_/*J*_0_ ∝ (1 − *t*^2^)^5/2^/(1 + *t*^2^)^1/2^ for *δl* pinning mechanism, while *J*_*c*_/*J*_0_ ∝ (1 − *t*^2^)^7/6^/(1 + *t*^2^)^5/6^ applies to *δT*_c_ pinning, where *t* = *T*/*T*_c_. Figure 8(a–f) shows almost perfect matching of the experimentally obtained *J*_*c*_ with the theoretically calculated values and favour *δl* pinning mechanism in the entire pressure region. Our analysis directly supports *δl* pinning mechanism whatever be the pressure, and it is responsible for increasing in *T*_c_ Our results suggest that there is a single vortex pinning due to spatial variations in the charge-carrier mean free path. The coherence length is proportional to the mean free path of the carriers, and therefore, the application of pressure leads to enhancement of *δl* pinning mechanism. One can note that similar results have been reported in case of Fe_x_NbSe_2_^6,38^, FeTe_0.7_Se_0.3_^61^ and pnictides^34^ superconducting compounds.

We have calculated the pinning force (*F*_p_ = *J*_c_*H*) as a function of magnetic field under various hydrostatic pressures and have investigated the magnetic field dependence of the pinning force density (*F*_p_) in order to understand vortex pinning mechanisms in this sample. Field dependence of F_p_ [Fig. S4] for various temperatures in superconductors may be scaled into a unique curve if they are plotted as a function of reduced field, *h* = *H*/H_irr_. The scaling of normalized pinning force from the Dew-Hughes formula^62^, *f*_*p*_ = *h*^*p*^(1 − *h*)^*q*^, where p and q are the parameters describing the pinning mechanism. Further, *J*_c_^0.5^*H*^0.25^ is plotted as a function of magnetic field is known as Kramer plot^63^ and it is used to determine *H*_irr_, where *H*_irr_ is determined as the extrapolated to zero field from various isotherms of *J*_c_ and the temperature dependent of *H*_irr_ shows a linear behavior.

**Figure 9:**
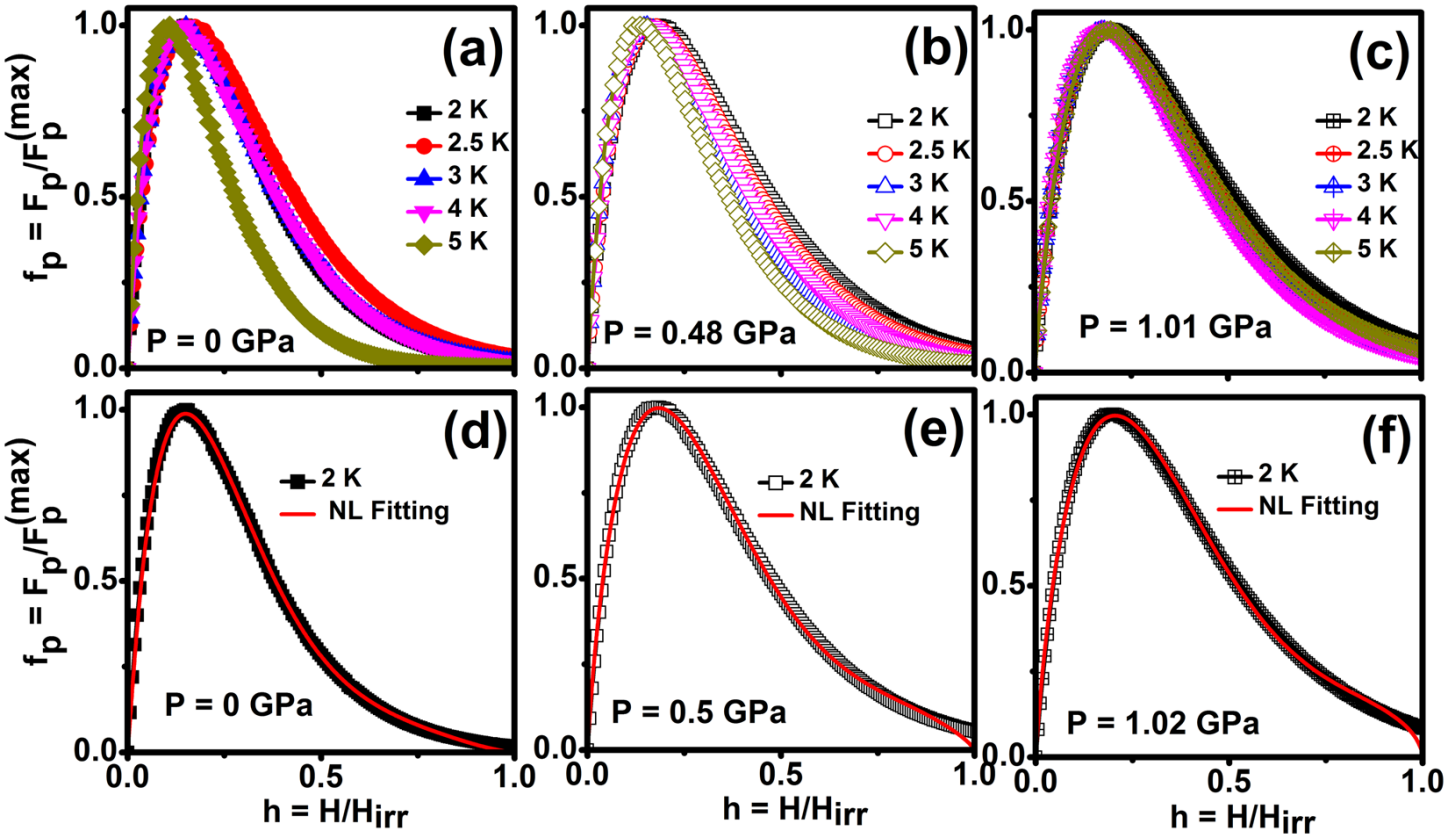
(**a**–**f**) Normalized pinning force density (*f*_p_ = *F*_p_/*F*_p_^(max)^) as a function of reduced magnetic field (*h* = *H*/*H*_irr_) for various hydrostatic pressures on Cr_0.0005_NbSe_2._ Solid lines are shown in the collective pinning model fitting at the log scale at a temperature of 2 K.

Figure 9(a–f) shows normalized pinning force (*f*_p_ = *F*_p_/*F*_p_^(max)^) as a function of reduced critical field (*h* = *H*/*H*_irr_) for various temperatures and constant *P* (0,0.5 and 1.02 GPa) [Fig. S5]. For the scaling of both point and surface pinning, we use the relation, *f*_*p*_ = *Ah*^*p*^(1 − *h*)^*q*^, where *p* and *q* describes the nature of specific pinning mechanism^62^. It is known from Dew-Hughes model, when *p* = 1/2 and *q* = 2 and *p* = 1 and *q* = 2 describes the surface pinning and point pinning respectively as predicted by Kramer^63^. The best fit of the curves is obtained with *f*_p_(*h*) dependence given by, *h*^0.89^(1 − *h*)^4.38^ + *Bh*^0.72^(1 − *h*)^1.38^ at 0 GPa and *h*^0.91^(1 − *h*)^5.70^ + *Bh*^0.79^(1 − *h*)^2.48^ at 1.02 GPa. The application of pressure causes an increase of *q* parameter from 4.38 to 5.70 which is an indication of the increase of both pinning center and surface pinning mechanism. It reveals that the normal core point pinning is more dominant than surface pinning mechanism under high pressure [Fig. S5]. The broadening of the pinning force with the application of pressure indicates that the pinning centers are enhanced with the application of pressure for this sample. Cr intercalation of into Se-Nb-Se layers of NbSe_2_ single crystals leads to an increase of more pinning centers compared with the undoped NbSe_2_.

## Conclusion

In summary, we have shown that hydrostatic pressure is a very effective means to significantly enhance *T*_c_, *J*_c_, *H*_irr_, and flux pinning in the Cr_0.0005_NbSe_2_ superconductor. We demonstrate that hydrostatic pressure significantly increases *T*_c_ from *M*(*T*) (d*T*_c_/d*P* = 0.91 K/GPa; 0 < *P* < 1 GPa) and ρ(*T*) (0.75 K/GPa; *P* < 1.5 GPa & 0.09 K/GPa; 1.5 < *P* < 8 GPa) measurements. The pressure introduces more point defects in sample and it is responsible for enhancement *J*_*c*_. We found that the hydrostatic pressure stabilizes a strong *δl* pinning mechanism. In addition, we found that the point pinning is more dominant than surface pinning under high pressure. The pressure enhanced the *H*_*c1*_, *H*_*c2*_, *H*_*irr*_ and reduces both coherence length and penetration depth which are responsible for the pinning mechanism.

## Experimental Techniques

Cr_0.0005_NbSe_2_ single crystal has been synthesized using chemical vapour transport method. The essential commercially available high purity elemental metallic powders such as Nb (99.95%), Se (99.99%) and Cr (99.99%) procured from Alfa Aeser, which are mixed with suitable stoichiometric ratio and made of 6 mm (*φ*) pellet. The pellet kept in a sealed quartz tube with tiny amount of iodine in the presence of liquid nitrogen atmosphere. The sealed quartz tube is placed in a two-zone furnace with programmable temperature controller and the temperatures of charge and growth zone at 800 °C and 720 °C respectively for a period of seven days. The grown crystals are characterized structural phase and the elemental composition analyses are confirmed using x-ray diffraction and Energy dispersive X-ray Spectroscopy techniques respectively and further details about synthesis and characterization of these materials were recently reported by Rukshana Pervin *et al*.^64^. A cubic anvil pressure device, consisting of six tungsten carbide (WC) anvils, which have been used to produce homogeneous hydrostatic pressure up to 8 GPa for resistivity measurements^65^. The applied pressure is calibrated from the resistance changes of Bi with its phase transitions at room temperature such as Bi I-II (2.55 GPa), Bi II-III (2.77 GPa) Bi III’-V (7.68 GPa) and Daphne #7373 used as a pressure transmitting medium^66^. The temperature of sample inside the Teflon cell is monitored by measuring the resistivity of three calibrated Pt(Co) resistance thermometers attached to the neck of each anvil. The electrical contacts were made using gold wire of 20 µm φ and Ag paste is used to make contacts on the surface of the sample with the typical sample size is 0.8 × 0.5 × 0.5 mm^3^. The standard four probe method is used for doing resistivity measurements under ambient and high pressure upto 8 GPa and the anvils are through the gold foil. The magnetic properties (ZFC & FC) of Cr_0.0005_NbSe_2_ single crystals were investigated under various magnetic fields at ambient and high pressures. The dc magnetization measurements under pressure were carried out using Physical Property Measurements System – Vibrating Sample Magnetometer (PPMS-VSM, Quantum Design, USA). The external pressure was generated up to ~1 GPa by a clamp type miniature hydrostatic pressure cell which is made of specially heat treated nonmagnetic Cu-Be alloy. The fluorinert FC #70 and FC #77 (1:1) mixture was used as a pressure transmitting medium and the *in-situ* pressure was estimated from the superconducting transition temperature of pure Sn which was loaded along with the sample in the capsule.

## Electronic supplementary material

Supplementary Information
