# Supplementary material for: Enhancement of superconducting properties and flux pinning mechanism on Cr0.0005NbSe2 single crystal under Hydrostatic pressure
Source: Sci Rep. 2019 Jan 23;9:347. doi: 10.1038/s41598-018-36672-x (PMC6344477; doi:10.1038/s41598-018-36672-x)
Supplement: Supplementary file 1 — Supplementary Information [file 41598_2018_36672_MOESM1_ESM.pdf]

Enhancement of superconducting properties and flux pinning mechanism on $Cr_{0.0005}NbSe_2$ single crystal under Hydrostatic pressure

S. Arumugam[a,*,$], Manikandan Krishnan[a,$], Kent Ishigaki[b], Jun Gouchi[b], Rukshana Pervin[c], G. Kalai Selvan[d], Parasharam M. Shirage[c] and Y. Uwatoko[b]

[a]Centre for High Pressure Research, School of Physics, Bharathidasan University, Tiruchirappalli 620024, India.

[b]Institute of Solid State Physics, University of Tokyo, 5-1-5 Kashiwanoha, Kashiwa, Chiba, 277-8581, Japan.

[c]Discipline of Metallurgy Engineering and Materials Science & Physics, Indian Institute of Technology Indore, Simrol Campus, Khandwa road, Indore 453552, India.

[d]Department of Physics, University of Alabama at Birmingham, Birmingham, AL, 35294, USA.

[$]These authors contributed equally to this manuscript.

[*] E-mail: sarumugam1963@yahoo.com

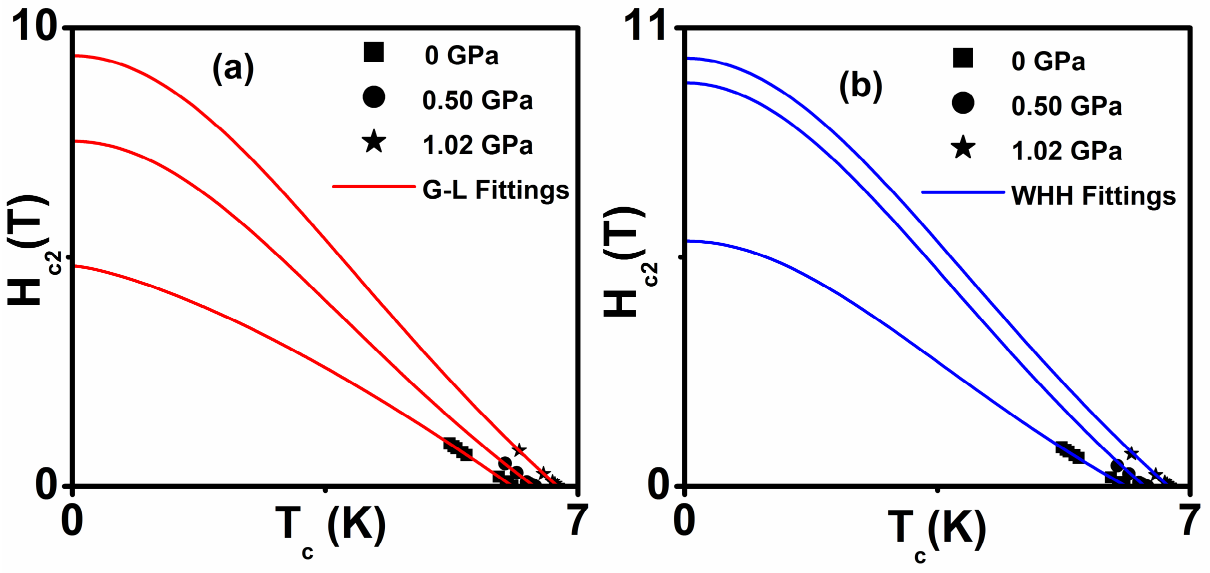

Fig. S1: (a) Superconducting transition temperature (T_c) dependent upper critical field (H_{c2}) for $Cr_{0.0005}NbSe_2$ sample at various hydrostatic pressures. Solid lines represent to fit the Ginzburg–Landau equation, (b) H_{c2} as a function of T_c for $Cr_{0.0005}NbSe_2$ sample at various pressures. Solid lines extrapolated $H_{c2}^{orb}(0)$ to fit the WHH equation.

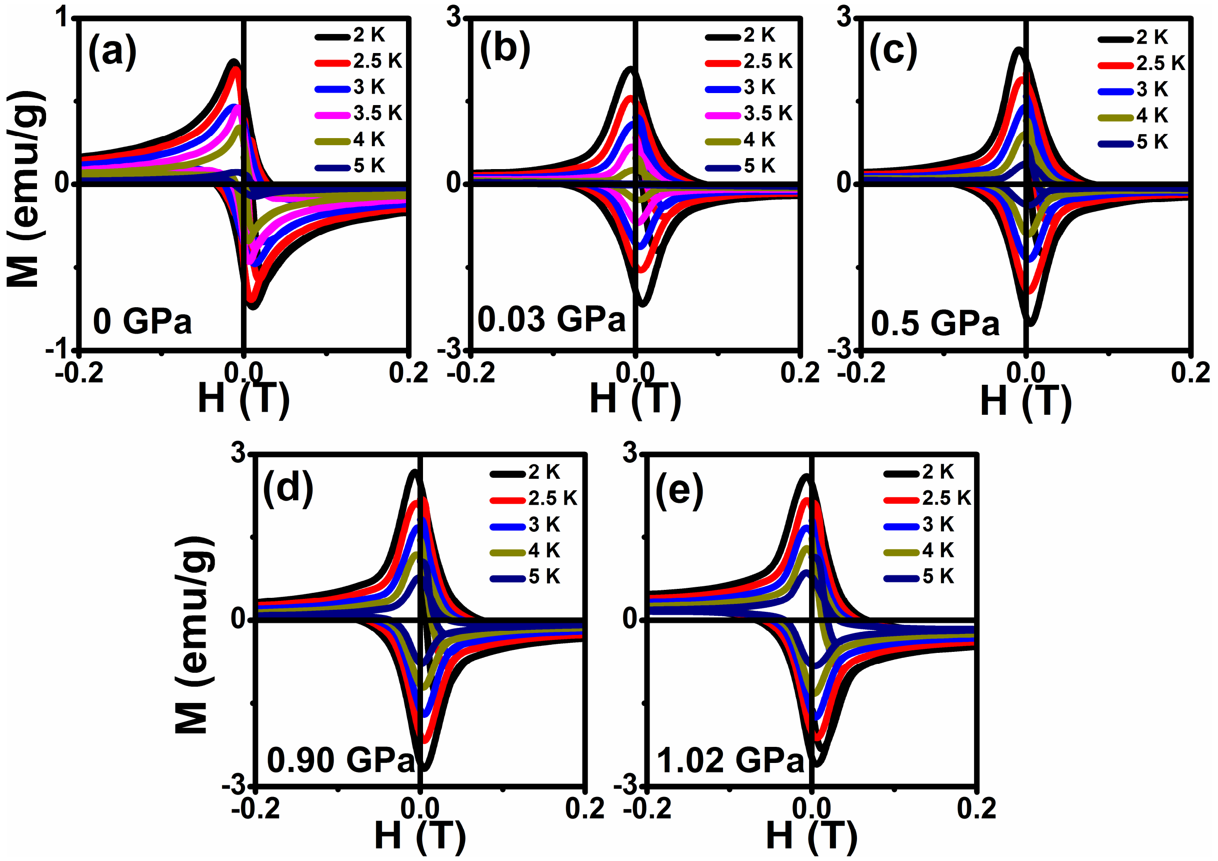

Fig. S2: (a), (b), (c), (d) and (e) Field dependent of magnetization scan in a low field range (dH/dt ~ 20 Oe/s) at various temperatures for 0 GPa, 0.03 GPa, 0.50 GPa, 0.90 GPa and 1.02 GPa respectively on $Cr_{0.0005}NbSe_2$.

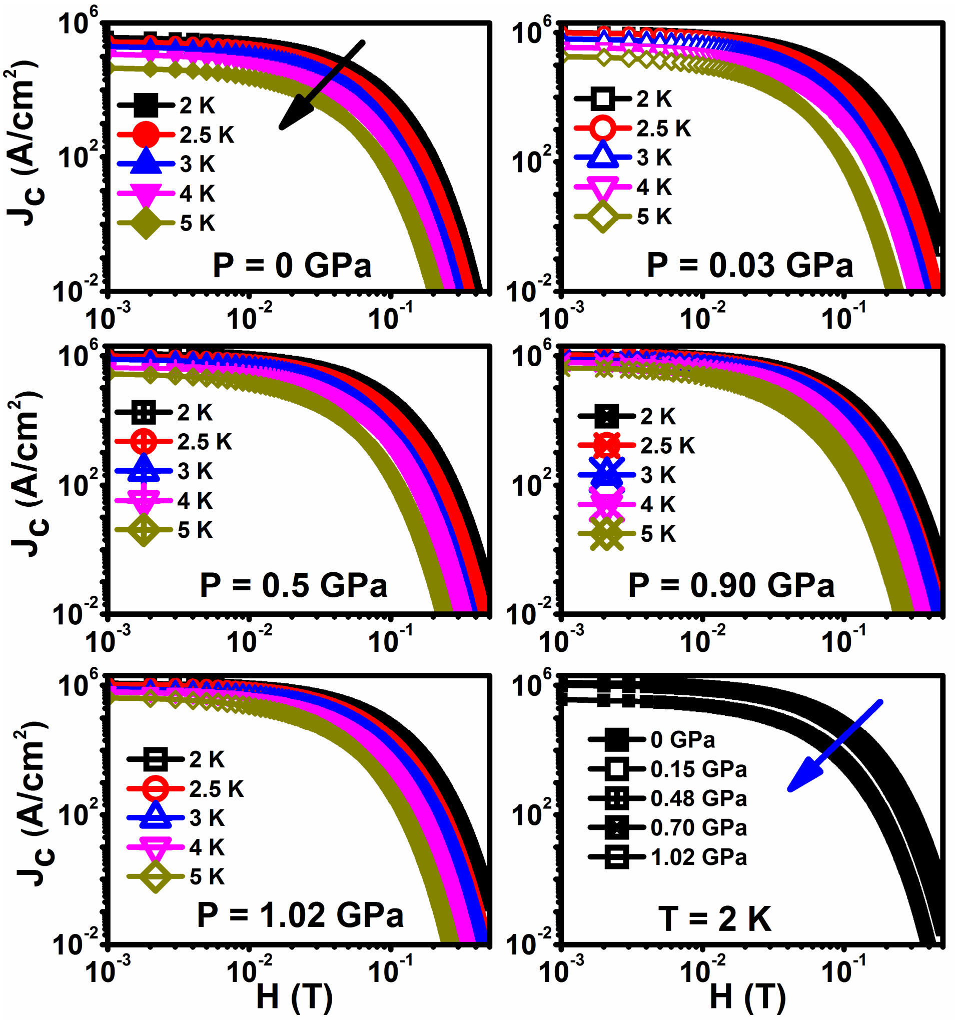

Fig. S3: Logarithmic scale of magnetic field dependent superconducting critical current density J_c(H) on $Cr_{0.0005}NbSe_2$ for various temperatures and few fixed hydrostatic pressures.

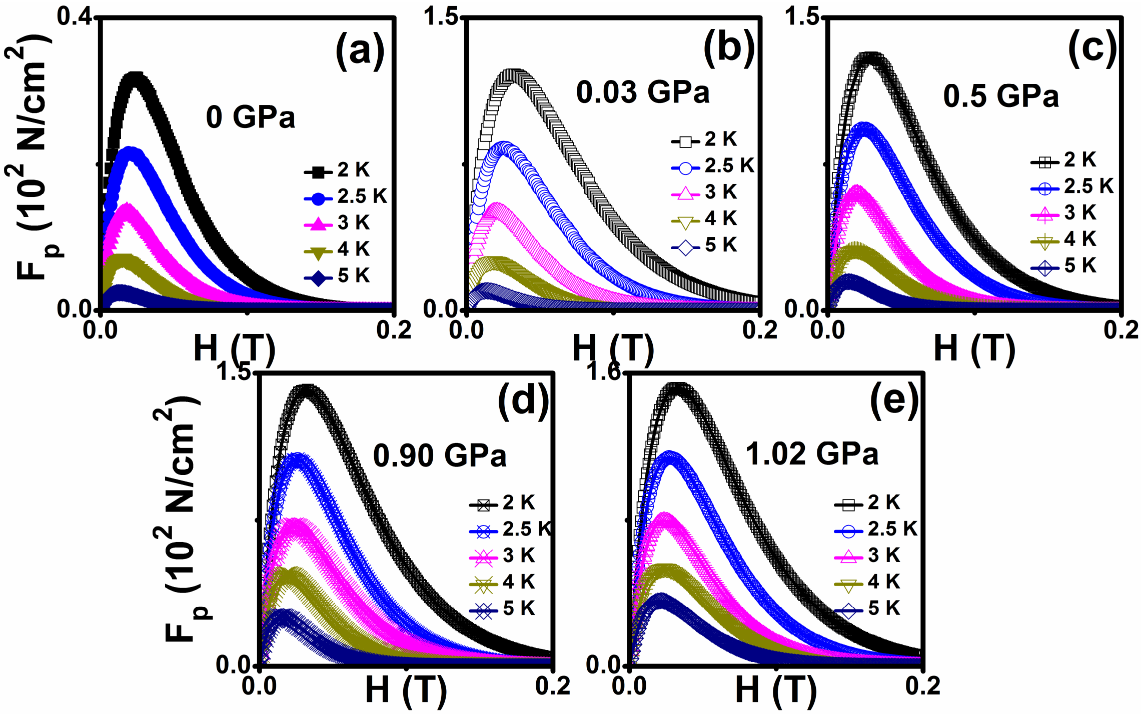

Fig. S4: Pinning force as a function of magnetic field for various hydrostatic pressures and temperatures on $Cr_{0.0005}NbSe_2$.

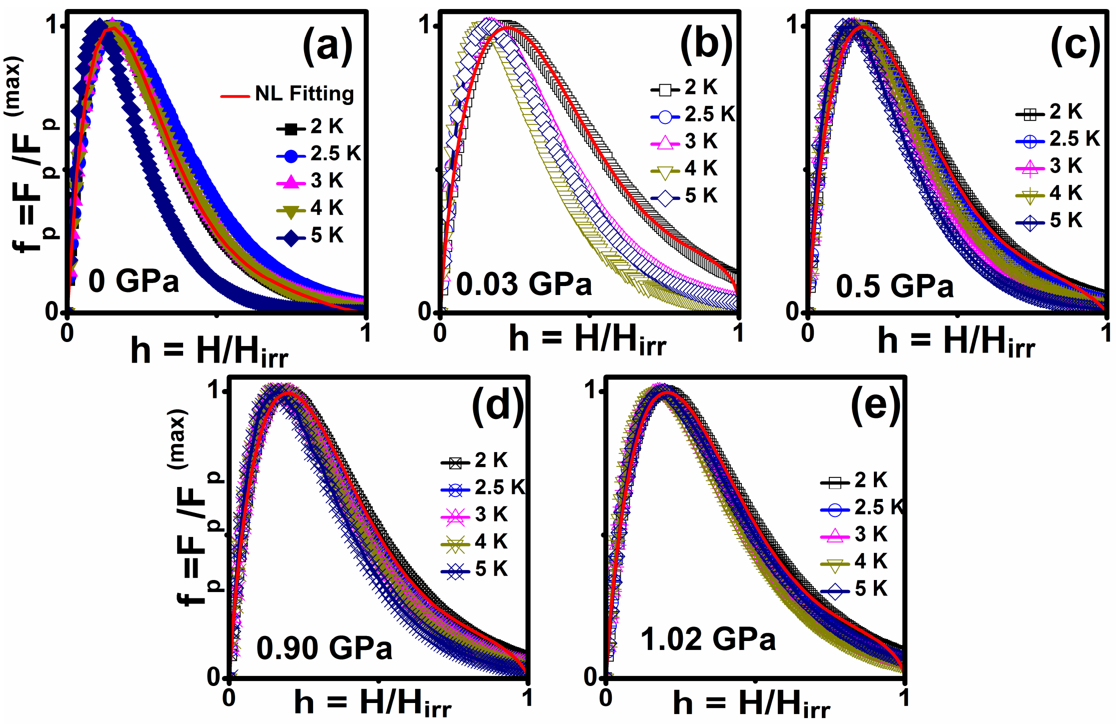

Fig. S5: Normalized pinning force density ($f_p = F_p/F_p^{(max)}$) as a function of reduced magnetic field (h=H/H_{irr}) for various hydrostatic pressures on $Cr_{0.0005}NbSe_2$. Solid lines are shown in the collective pinning model fitting.
